# Artificial intelligence meets pediatric orthopedics: A comparative analysis of ChatGPT-4o, Gemini 2.0, and Claude 3.5 in detecting supracondylar humeral fractures

**DOI:** 10.1371/journal.pone.0353782

**Published:** 2026-07-14

**Authors:** Utku Murat Kalafat, Hüseyin Mutlu, Ramiz Yazıcı, Murat Genç, Bensu Bulut, Medine Akkan Öz, Ayşenur Gür, Mehmet Yortanlı, Uğur Şakar

**Affiliations:** 1 Department of Emergency Medicine, İstanbul Kanuni Sultan Süleyman Training and Research Hospital, University of Health Sciences, İstanbul, Türkiye; 2 Department of Emergency Medicine, Aksaray University Medical School, Aksaray, Türkiye; 3 Department of Emergency Medicine, Ankara Training and Research Hospital, Ankara, Türkiye; 4 Department of Emergency Medicine, Gülhane Training and Research Hospital, University of Health Sciences, Ankara, Türkiye; 5 Department of Emergency Medicine, Etimesgut Şehit Sait Ertürk State Hospital, Ankara, Türkiye; 6 Department of Emergency Medicine, Konya Numune Hospital, Konya, Türkiye; 7 Department of Emergency Medicine, Dışkapı Yıldırım Beyazıt Training and Research Hospital, Ankara, Türkiye; China University of Petroleum East China - Qingdao Campus, CHINA

## Abstract

**Background:**

Supracondylar humeral fractures constitute 10–16% of pediatric skeletal injuries, requiring timely diagnosis to prevent neurovascular complications. Developmental variations in pediatric bone structures pose diagnostic challenges for clinicians. This study evaluated three next-generation large language models (LLMs) (ChatGPT-4o, Gemini 2.0, Claude 3.5) for detecting pediatric supracondylar humeral fractures and their classification according to the Gartland system.

**Methods:**

This retrospective observational study included 300 pediatric patients (150 with supracondylar humeral fractures confirmed by expert consensus, 150 without fractures) aged 2–10 years presenting to the Emergency Department of the Bilkent City Hospital (October 2022-January 2025). Two-view elbow radiographs were presented to each LLM three times on different days. Diagnostic accuracy was evaluated using overall accuracy (all three responses correct), strict accuracy (≥2 correct responses), and ideal accuracy (≥1 correct response). Response consistency was assessed using Fleiss’ Kappa coefficient. Fractures were classified according to modified Gartland criteria.

**Results:**

Gemini 2.0 demonstrated highest sensitivity (68.4%) followed by Claude 3.5 (58.7%) and ChatGPT-4o (19.3%) for fracture detection (p < 0.001). Ideal accuracy rates were 83.3%, 78.7%, and 27.3% respectively. Although ideal accuracy rates exceeded 91% in non-fracture cases, specificity remained low (33.1–36.0%), indicating a high rate of false-positive classifications. Response consistency was very good for ChatGPT-4o (κ = 0.69) and Gemini 2.0 (κ = 0.61), good for Claude 3.5 (κ = 0.44). For Gartland classification, Gemini 2.0 achieved highest accuracy: Type I (83.3%), Type II (62.4%), Type III (68.7%).

**Conclusion:**

Current LLMs demonstrate limited capability as independent diagnostic tools for pediatric supracondylar humeral fractures. Gemini 2.0’s 68.4% sensitivity indicates these technologies require specialized pediatric training before clinical implementation. However, their potential as assistive tools for triage and assessment warrants further development of pediatric-specific models.

## 1. Introduction

Supracondylar fractures of the humerus are one of the most common elbow injuries seen in the pediatric age group and accounts for approximately 10–16% of all childhood bone fractures [1]. This type of fracture occurs particularly in children aged 5–7 years, usually by falling on an outstretched hand [2]. Timely and accurate diagnosis is of critical importance, because delayed or inaccurate diagnosis can cause neurovascular complications, compartment syndrome and permanent functional losses [3]. Developmental properties of the bone structures in pediatric populations are one of the factors that makes diagnosis harder. Age-related changes in ossification centres, growth plates and anatomical variations cause diagnostic challenges for inexperienced clinicians [4]. The Gartland classification system is a system commonly utilised for assessing supracondylar fractures of the humerus and provides a standardized approach for treatment [5]. This classification categorises fractures from Type I to Type III based on the degree of displacement and offers specific treatment options for each category [6].

Application of artificial intelligence technologies in the medical field have caused a paradigm shift in diagnosis and treatment processes in recent years [7–9]. Deep learning algorithms and large language models (LLMs) offer promising results, particularly in interpreting radiological images [7, 10]. Current LLMs such as ChatGPT-4o, Gemini 2.0 and Claude 3.5 have the potential to interpret radiological images [11, 12]. Hirosawa et al.’s study showing improvement in diagnostic accuracy with the integration of visual data in ChatGPT-4 highlights the potential of these technologies in analysing radiologic images [13]. Similarly, a comprehensive study by Wang et al. performed on a data set of chest x-rays emphasises the critical role of large-scale data sets in educating artificial intelligence [14]. These developments illustrate the potential value artificial intelligence support can provide in specific diagnostic areas, particularly in areas such as supracondylar humeral fractures, where fine anatomical details are important.

These models show promise for utilization in the emergency department, especially for rapid assessment and triage [15], however, LLM applications in pediatric radiology is still limited. The existing literature mainly focuses on adult populations, and the unique characteristics of pediatric anatomy have not been sufficiently researched [16]. Considering the challenges in diagnosing pediatric supracondylar humeral fractures and the growing capabilities of artificial intelligence technologies, evaluating the diagnostic performances of LLMs in this area is of critical importance. We aimed to evaluate the diagnostic performance of ChatGPT-4o, Gemini 2.0 and Claude 3.5 in detecting pediatric supracondylar humeral fractures and to assess their ability to classify these fractures according to the Gartland classification.

## 2. Methods

### 2.1. Study design and Participants

This retrospective observational study was performed in the Emergency Department of the Bilkent City Hospital (October 2022-January 2025). Approval for this study was obtained from Bilkent City Hospital Clinical Research Ethics Committee (Ethics Committee date: March 5, 2025, Decision No: E2-25–10248). Access to the anonymized patient data for research purposes was obtained between March 6, 2025, and July 6, 2025. The emergency department our study was conducted in is a Level 1 trauma centre, servicing approximately 1000 trauma patients monthly.

Patients between the ages of 2–10 presenting to our trauma centre due to elbow injuries caused by traffic accidents, falls from heights and sports injuries, had two-way elbow x-rays taken, and whose images were accessible from the hospital’s electronic data system were included in our study. All patient data were anonymized and sequentially numbered prior to inclusion in the study, and no direct personal identifiers were used at any stage of the research. This study represents a retrospective analysis of previously collected, anonymized data and did not involve direct interaction with human participants. Therefore, individual consent to participate was not required. Ethical approval was obtained, and given the retrospective nature of the study, the ethics committee waived the requirement for informed consent. Patients with open fractures and/or fractures accompanied by dislocations, patients who had previously undergone surgery or treatment for elbow fractures or dislocations, those whose images could not be accessed from the hospital’s electronic data system, and patients under the age of 2 and over the age of 10 were excluded from the study. Images of the included patients were saved in PNG format with a resolution of 512 x 512 pixels after the removal of DICOM identifiers.This resolution was selected to standardize image inputs across all models and to ensure consistent processing conditions. In addition, no personally identifiable or sensitive patient data were entered into the LLMs, and all analyses were performed using fully anonymized data in accordance with data protection principles. Patients’ age, gender and complaint at the time of presentation, reference diagnosis and imaging parameters were recorded. Two-way elbow x-rays of patients were separated into two groups depending on whether they had supracondylar humeral fractures or not by the authors R.Y. (20 years emergency department experience) and H.M. (over 10 years orthopaedics experience). Additionally, patients with fractures were categorised as the following, based on the modified Gartland classification system:

Type I – Radiographic evidence of elbow effusion (posterior fat pad sign), non- displaced or minimally displaced (<2mm) fracture.

Type II – Displaced fracture (>2mm) with intact posterior periosteum.

Type III – Completely displaced fracture where both anterior and posterior periosteum is damaged. This injury refers to the absence of continuity between proximal and distal pieces.

In cases where the diagnosis and classification differed between authors, the final decision was made by the other author, B.B. (over 11 years of experience).

For more effective interpretation, the models were provided with contextual information based on orthopedic textbook content regarding supracondylar humeral fractures and their classification within the input prompts. No fine-tuning or model retraining was performed. The following model variants were used in this study: ChatGPT-4o (OpenAI, Pro version), Gemini 2.0 Flash (Google), and Claude 3.5 (Anthropic, Pro version). All models were accessed through their publicly available interfaces during the study period (May 2025). For each case, the anteroposterior and lateral elbow radiographs were uploaded as two separate image files. Following this, ChatGPT-4o, Gemini 2.0 and Claude 3.5 models were presented with the question “patient presented to the emergency department following trauma, has pain and/or swelling in their elbow, following are the images, does this patient have supracondylar fracture of the humerus or not” by author M.Y. on the same computer, once every day on three different days between 25–31 May 2025 and three responses were generated for each question. The same standardized prompt structure was used for all models and cases. The prompts included a brief clinical scenario and instructions to determine the presence of a fracture and, if present, to classify it according to the modified Gartland system. Each case was evaluated in a separate and independent chat session. After each evaluation, the conversation was terminated, and a new session was initiated for the subsequent case. No prior interactions were retained between cases, ensuring independent assessment and preventing potential context leakage. If an LLM responded with “yes, there is a fracture”, it was asked to classify it according the modified Gartland system and three responses were generated for each question. This sequential diagnostic workflow mirrors clinical practice, where facture detection is followed by classification, allowing both processes to be evaluated in a clinically meaningful manner.This approach is similar to other studies where LLMs were presented with questions three times to improve consistency and response stability [17–19]. Accuracy rates of models were assessed using the overall accuracy, strict accuracy and ideal accuracy criterion.

Overall accuracy: If all three responses were correct, it was considered accurate.

Strict Accuracy: If at least two out of three answers are correct, it was considered accurate.

Ideal accuracy: If at least one of the three answers was correct, it was considered accurate.

The unit of analysis differed according to the metric of interest. For sensitivity, specificity, positive predictive value (PPV), and negative predictive value (NPV), the unit of analysis was defined at the session (response) level. Each case was evaluated three times, and each response was treated as an independent observation in the calculation of TP, FP, TN, and FN values.

In contrast, overall accuracy, strict accuracy, and ideal accuracy were calculated at the case level using predefined aggregation rules (all three responses correct, at least two correct, and at least one correct, respectively).

### 2.2. Statistical analysis

Data obtained in this study was analysed using IBM SPSS Statistics Version 27.0 (IBM Corp., Armonk, NY, USA). Distribution of constant variables was first assessed with Shapiro-Wilk test. Constant variables not showing a normal distribution were identified as median and interquartile range (1^st^ quarter – 3^rd^ quarter), and for the comparison of the two groups Mann-Whitney U test was preferred among the non-parametric tests. Categorical variables were presented as frequency (n) and percentage (%); the difference between these variables were analysed using Pearson Ki-square test.

Diagnostic accuracy rates of the three different LLMs (ChatGPT-4o, Gemini 2.0 and Claude 3.5) were assessed under three different categories; “overall accuracy” (all three responses are correct), “strict accuracy” (at least 2 responses are correct), and “ideal accuracy” (at least one response is correct). Each model’s accuracy rate was analysed separately for the group with supracondylar humeral fracture and the non-fracture group. Models’ inter-group comparisons based on this accuracy criterion was done with Cochran’s Q test; in cases where significant difference were detected, post-hoc McNemar tests were performed for pairwise comparisons. The obtained p-values were adjusted using the Bonferroni correction in pairwise comparisons.

Moreover, the internal consistency of responses generated by each model for the same patient in three different sessions was evaluated using Fleiss’ Kappa coefficient. This analysis was performed separately for both the group with supracondylar humeral fracture and the non-fracture group. Fleiss’ Kappa coefficients, 95% confidence intervals and p values were calculated. Obtained Kappa values were interpreted as “weak” if in the 0.00–0.20 range, “moderate” if in the 0.21–0.40 range, “good” if in the 0.41–0.60 range and “very good” if in the 0.61 and above range. For all analysis, a two-tailed p < 0.05 value was considered statistically significant.

## 3. Results

In total 300 patients were included in the study. The group with a supracondylar humeral fracture and the non-fracture group were similar in age and gender distribution. When the distribution of the 150 patients in the supracondylar humeral fracture group was examined according the modified Gartland classification; 14 (9.3%) patients were classified as Type I, 39 (26%) as Type II and 97 (64.7%) as Type III (Table 1).

**Table 1:** Demographic data of groups with and without supracondylar fractures of the humerus.

Variables	Supracondylar fracture (n = 150)	Non-fracture (n = 150)	p
Age, years	5.6 (±3.1)	5.1 (±2.9)	0.357
Sex, n (%)			
Male	107 (71.3)	96 (64.0)	0.175
Female	43 (28.7)	54 (36.0)	
Gartland classification, n (%)			
Type I	14 (9.3)		
Type II	39 (26.0)		
Type III	97 (64.7)		

**Fig 1:**
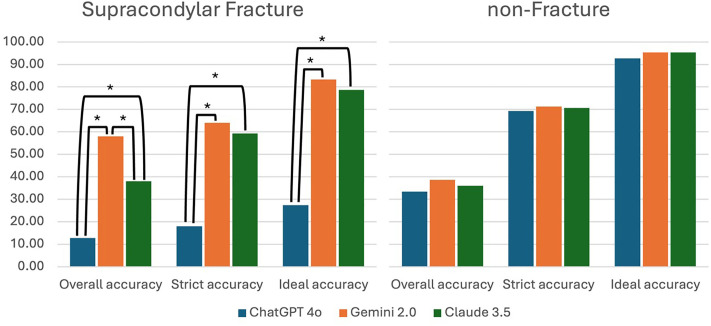
Comparison of accuracy rates of different artificial intelligence models in diagnosing supracondylar fractures.

When diagnostic accuracy of artificial intelligence models in cases with fractures was compared, it was determined that Gemini 2.0 and Claude 3.5 performed significantly better than ChatGPT-4o in terms of overall accuracy (58.0% and 38.0% vs 12.7%), strict accuracy (64.0% and 59.3% vs 18.0%) and ideal accuracy (83.3% and 78.7% vs 27.3%) (p < 0.001). In the non-fracture group, overall accuracy was between 32.7–38.0%, strict accuracy was between 68.0–70.7% and ideal accuracy was between 91.3–93.3%, and there was no significant difference between models in terms of accuracy (p > 0.05) (Table 2, Fig 1).

**Table 2:** Comparison of diagnostic accuracy rates of artificial intelligence models between the supracondylar fracture group and the non-fracture group.

	ChatGPT 4o	Gemini 2.0	Claude 3.5	p
*Supracondylar fracture (n = 150);*				
Overall accuracy	19 (12.7)	87 (58.0)	57 (38.0)	<0.001
Strict accuracy	27 (18.0)	96 (64.0)	89 (59.3)	<0.001
Ideal accuracy	41 (27.3)	125 (83.3)	118 (78.7)	<0.001
*Non-fracture (n = 150);*				
Overall accuracy	49 (32.7)	57 (38.0)	55 (36.7)	0.538
Strict accuracy	102 (68.0)	103 (68.7)	106 (70.7)	0.793
Ideal accuracy	137 (91.3)	139 (92.7)	140 (93.3)	0.727

Values marked with * indicate statistically significant differences.

In the post-hoc McNemar analyses conducted after the Cochran’s Q test, the Gemini 2.0 (p < 0.001) and Claude 3.5 (p < 0.001) models demonstrated significantly higher accuracy than the ChatGPT-4o model, and the Gemini 2.0 (p < 0.001) model demonstrated significantly higher accuracy than the Claude 3.5 model. In terms of overall accuracy and ideal accuracy criterion, both the Gemini 2.0 (p < 0.001) and Claude 3.5 (p < 0.001) models demonstrated significantly higher accuracy than the ChatGPT-4o model.

**Fig 2:**
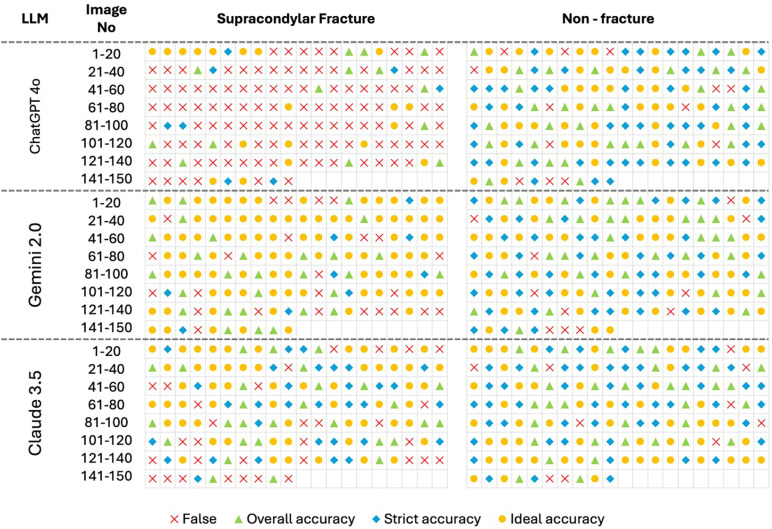
Distribution of artificial intelligence models based on response accuracy categories.

Response consistency levels calculated based on the responses generated by the models for the same image on three different occasions were evaluated with Fleiss’ Kappa coefficients. In the group with supracondylar humeral fracture, ChatGPT-4o (κ = 0.69; 95% GA: 0.59–0.78) and Gemini 2.0 (κ = 0.61; 95% GA: 0.52–0.70) showed very good consistency and Claude 3.5 (κ = 0.44; 95% GA: 0.35–0.53) showed statistically significant good consistency (p < 0.001). In the non-fracture group, all models showed weak consistency; ChatGPT-4o (κ = 0.15; 95% GA: 0.06–0.24; p = 0.001), Gemini 2.0 (κ = 0.18; 95% GA: 0.09–0.27; p < 0.001) and Claude 3.5 (κ = 0.15; 95% GA: 0.06–0.24; p = 0.002) showed weak consistency (Fig 2.)

All models responded to 450 images with supracondylar humeral fractures and 450 images without fractures. From the images with fracture, Gemini 2.0 categorised 68.4% (n = 308) accurately and Claude 3.5 58.7% (n = 264), while ChatGPT-4o categorised only 19.3% (n = 87) accurately. On the other hand, in the non-fracture group classification accuracy was much higher and closer to each other (ChatGPT-4o: 64.0%; Gemini 2.0: 66.7%; Claude 3.5: 66.9%).

Analysis of models’ performance based on the responses generated for all 900 images, showed that Gemini 2.0 had the highest success rate in detecting supracondylar humeral fractures, with 68.4% sensitivity and 50.7% positive predictive value (PPV). Claude 3.5 also showed a similar performance, with 58.7% sensitivity and 46.7% PPV. On the hand, ChatGPT-4o was significantly less successful than the other models, with a rather low sensitivity of 19.3% and 23.2% PPV. In the cases without fracture, specificity was low in all models. In terms of negative predictive value (NPV), there was no statistically significant difference between models (Table 3). 95% confidence intervals for sensitivity, specificity, positive predictive value (PPV), and negative predictive value (NPV) were calculated to provide a more robust estimation of diagnostic performance.

**Table 3:** Diagnostic performance criterion of three large language models in diagnosing supracondylar fractures.

	TP	FP	TN	FN	Sensitivity	Specificity	PPV	NPV
**ChatGPT 4o**	87	288	162	363	19.3	36.0	23.2	30.9
**Gemini 2.0**	308	300	150	142	68.4	33.3	50.7	51.4
**Claude 3.5**	264	301	149	186	58.7	33.1	46.7	44.5

**Abbreviations: TP**: True positive; **FP**: False positive; **FN**: False negative; **TN**: True negative; **PPV**: Positive predictive value; **NPV**: Negative predictive value.

**Fig 3:**
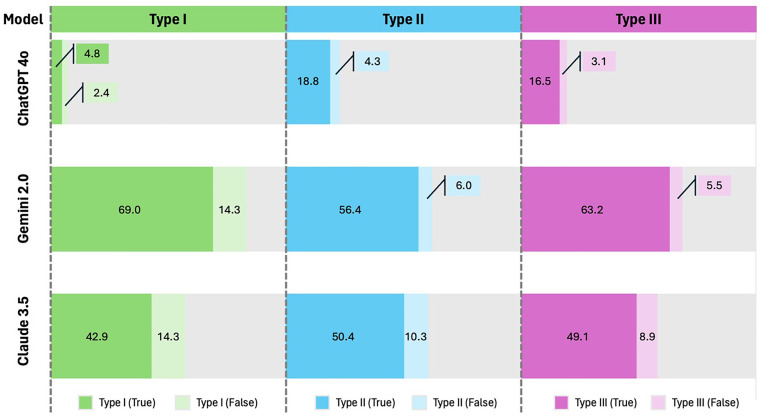
Response distribution of artificial intelligence models based on the modified Gartland classification.

In the supracondylar humeral fracture group, models were asked to classify the images with fractures according to the modified Gartland classification system. For the cases with Type 1 fracture, fracture recognition rate of ChatGPT-4o was 7.1% (n = 3), of Gemini 2.0 it was 83.3% (n = 35) and of Claude 3.5 it was 57.1% (n = 24). When they detected fractures, correct Type 1 classification rate was 66.7% (n = 2) for ChatGPT-4o, 82.9% (n = 29) for Gemini 2.0 and 75.0% (n = 18) for Claude 3.5. For Type II fractures, correct detection rate was 23.1% (n = 27) for ChatGPT-4o, 62.4% (n = 73) for Gemini 2.0 and 60.7% (n = 71) for Claude 3.5. In cases where fractures were detected, correct classification was made in 81.5% (n = 22) of cases by ChatGPT-4o, in 90.4% (n = 66) of cases by Gemini 2.0 and in 83.1% (n = 59) of cases by Claude 3.5. For cases with Type III fractures, ChatGPT-4o detected fractures in 19.6% (n = 57) of cases, Gemini 2.0 in 68.7% (n = 200) and Claude 3.5 in 58.1% (n = 169). Among the cases they detected fractures in, the rates for correct classification as Type III was 83.1% (n = 59) in ChatGPT-4o, 92.0% (n = 184) in Gemini 2.0 and 84.6% (n = 143) in Claude 3.5 (Fig 3).

*Shows the rates of accurate detection and classification of fractures of ChatGPT-4o, Gemini 2.0, and Claude 3.5 models according to the Gartland classification. Coloured sections: Models’ accurate fracture detection rates; Dark coloured sections: Models’ accuracy classification rates; Light coloured sections: Models’ classification error rates.*

## 4. Discussion

Rapidly developing abilities of artificial intelligence technologies in the field of medical image analysis creates new opportunities in radiology practises and offers promising results in the improvement of diagnostic accuracy. Our study demonstrates that LLMs show significant variability in the diagnosis of pediatric supracondylar fractures of the humerus. We have determined that Gemini 2.0 (68.4% sensitivity, 50.7% PPV) and Claude 3.5 (58.7% sensitivity, 46.7% PPV) performed significantly better when compared to ChatGPT-4o (19.3% sensitivity, 23.2% PPV) in images with fractures. On the other hand, specificity was found to be similarly low for all models, with 36% for ChatGPT-4o, 33.3% for Gemini 2.0 and 33.1% for Claude 3.5. In addition, the positive predictive value (PPV) was low across the models, particularly for ChatGPT-4o, indicating that a substantial proportion of predicted fractures were false positives. Clinically, this may result in unnecessary imaging, overdiagnosis, and increased workload in emergency departments, thereby limiting the real-world applicability of these systems. While ideal accuracy rates were high in non-fracture cases, this metric may overestimate model performance, as it is based on at least one correct response out of three attempts and does not adequately reflect false-positive rates. In binary classification settings, this approach increases the likelihood of obtaining at least one correct prediction, even when false-positive rates remain high. Therefore, ideal accuracy should be interpreted cautiously alongside specificity. The low specificity observed in our study indicates a substantial tendency of the models to incorrectly classify healthy cases as fractures, representing an important limitation for clinical applicability. This discrepancy is further explained by differences in the level of analysis. While ideal accuracy is calculated at the case level and reflects at least one correct response across repeated attempts, specificity is calculated at the session level and reflects the frequency of false-positive predictions. Therefore, specificity provides a more conservative and clinically relevant estimate of diagnostic reliability. In the Gartland classification, Gemini 2.0 was the most successful with accuracy rates between 62.4–83.3%. This finding should be interpreted within the context of the sequential evaluation applied in our study, where fracture detection was followed by classification. This workflow reflects routine clinical practice and enables a combined interpretation of detection and classification performance. To our knowledge, our study is the first comprehensive study comparing the three LLMs’ (ChatGPT-4o, Gemini 2.0 and Claude 3.5) diagnostic performances in detecting supracondylar fractures of the humerus in pediatric patients.

When performing a detailed analysis of ChatGPT-4o, Gemini 2.0 and Claude 3.5, our study showed dramatic differences, particularly in diagnosing fractures. The 19.3% sensitivity of ChatGPT-4o in cases with supracondylar fractures, compared to the 68.4% sensitivity of Gemini 2.0 and 58.7% of Claude 3.5, demonstrates the significance of model architecture and training data. In their study evaluating GPT-4 V’s performance in identifying radiological findings in chest radiographs, Zhou et al. also found similar variations between the performances of different models [17]. The poor performance of ChatGPT-4o found in our study is consistent with Horiuchi et al.’s musculoskeletal radiology study showing that ChatGPT had an accuracy rate of 43% when working with text-based information and that this rate dropped to 8% when working with image-based information [18], and Huppertz et al.’s study on radiological images reporting that the diagnostic accuracy rate of GPT-4V is 8.3% [19]. The poor performance of ChatGPT-4o in our study indicates that ChatGPT-4o’s limitations in image processing continue.

Similarly, previous studies evaluating the diagnostic performance of artificial intelligence in fracture detection have demonstrated that model performance may vary depending on fracture type and anatomical complexity. For instance, Bulut et al. reported relatively high diagnostic performance of LLMs in detecting scaphoid fractures [20]. In our study, supracondylar humeral fractures were selected due to their clinical importance as one of the most common pediatric elbow fractures requiring surgical intervention. The relatively higher diagnostic performance observed in certain models may be related to the characteristic radiographic features of these fractures, similar to findings reported in prior studies.

In their study evaluating ChatGPT-4’s performance in orthopaedic foot and ankle pathologies, Essis et al. reported that the model can effectively diagnose simple bone related cases but struggled to provide comprehensive information [21]. Similarly, recent studies have reported that artificial intelligence models generate more robust responses to easier questions and worse responses to more difficult questions [22,23]. The fact that Gemini 2.0, Claude 3.5 and ChatGPT-4o performed significantly better in cases without supracondylar fractures of the humerus (66.7%, 66.9% and 64.0%, respectively) in our study, when compared to cases with fracture (68.4%, 58.7% and 19.3%, respectively), demonstrates that these models are more successful when radiologic findings are clear and normal.

Noda et al. reported that artificial intelligence was successful in classifying pertrochanteric fractures of the femur in their study [24]. In our analysis of sub-groups based on the Gartland classification of supracondylar humeral fractures, we also found that the accurate diagnosis rate of Gemini 2.0 is 83.3% in Type I fractures. This high success rate in detecting fractures with minimal displacement indicates that this model has effective pattern recognition abilities. On the other hand, the 7.1% success rate of ChatGPT-4o in Type I fractures shows that this model has difficulty detecting radiographic findings.

Shultz et al. have reported kappa values between 0,67 and 0,79 among experienced radiologists in their study evaluating the reliability of the Gartland classification in paediatric supracondylar humeral fractures [25]. Similarly, ChatGPT-4o (κ = 0,69) and Gemini 2.0 (κ = 0,61) have shown very good consistency in the group with supracondylar humeral fracture, whereas Claude 3.5 (κ = 0,44) have shown good consistency, demonstrating the significant difference between models in reliability. Horiuchi et al. have also reported similar consistency issues in their study where GPT-4V displayed worse diagnostic performance than GPT-4 in neuroradiology cases [18]. Moreover, poor consistency shown by all models in the non-fracture group (κ = 0,15−0,18) indicate that LLMs experienced greater uncertainty in interpreting negative findings. Furthermore, the discrepancy in kappa values between fracture and non-fracture cases suggests that the models were more consistent in identifying fractures than normal findings. The markedly lower agreement in non-fracture cases indicates a tendency to overpredict fractures, reflecting a systematic bias that may contribute to the high false-positive rates and limit clinical reliability.

Compared to previously published convolutional neural network (CNN)-based models developed specifically for radiographic fracture detection, the performance of LLMs in our study appears lower, particularly in terms of specificity (33.1–36.0%). Previous studies using dedicated imaging models have reported substantially higher diagnostic performance, with sensitivity and specificity values often exceeding 85–90% in musculoskeletal fracture detection tasks [26]. Additionally, CNN-based systems have demonstrated robust performance in real-world clinical settings, including automated detection, classification, and localization of fractures with external validation [27]. This difference is further influenced by the nature of training data and task optimization. CNN-based models are trained exclusively on large-scale, high-quality annotated radiographic datasets, allowing them to learn subtle and domain-specific imaging features critical for fracture detection. In contrast, general-purpose LLMs are trained on heterogeneous multimodal data and lack dedicated optimization for fine-grained visual pattern recognition. As a result, LLMs may have limited sensitivity in detecting subtle radiographic abnormalities and may exhibit a higher tendency toward false-positive predictions. CNN-based systems are specifically trained on large, annotated radiographic datasets and optimized for visual feature extraction, whereas LLMs are general-purpose multimodal models that are not specifically optimized for radiographic interpretation. Accordingly, LLMs should not be considered as substitutes for specialized radiology AI systems. Rather, they may serve as supportive, general-purpose tools that can assist clinicians in preliminary assessment and clinical reasoning.

Although LLMs demonstrated certain diagnostic capabilities in this study, they cannot currently replace clinical decision-making by experienced physicians. Their performance, particularly in terms of sensitivity and specificity, remains insufficient for independent use in real-world emergency settings. Therefore, these systems should be considered as supportive tools that may assist clinicians rather than substitute them. Future studies comparing the performance of AI systems with that of experienced clinicians may provide more clinically relevant insights.

Our study demonstrates that the latest versions of LLMs (ChatGPT-4o, Gemini 2.0 and Claude 3.5) have made significant strides in interpreting images using real patient data. Evaluating a large number of images with and without pediatric supracondylar fractures of the humerus and classifying these fractures based on the Gartland classification using LLMs are the strengths of our study. However, our study also has limitations. Firstly, patient selection might potentially be biased due to the retrospective design. Secondly, our study has used data from a single centre, and the performance of artificial intelligence models might have to be verified in different populations and geographical locations. Differences in imaging protocols, patient demographics, and disease presentation across institutions may influence model performance and limit generalizability. Therefore, multicenter studies are needed to confirm the robustness and clinical applicability of these models. Another important limitation of this study is the use of downscaled images (512x512 pixels), which may have reduced the visibility of subtle radiographic findings, particularly in minimally displaced (Gartland Type I) fractures. The use of higher-resolution images may improve model performance and should be considered in future studies. Additionally, the absence of a direct comparison between AI model performance and that of experienced clinicians may limit the interpretation of their clinical applicability. Finally, another limitation of this study is the rapid evolution of LLMs. The models evaluated reflect their performance at the time of data collection (May 2025), and newer versions released thereafter may demonstrate different capabilities. Therefore, the findings should be interpreted within this temporal context.

## 5. Conclusion

In conclusion, this study evaluating the performances of artificial intelligence models in diagnosing pediatric supracondylar fractures of the humerus, have shown that Gemini 2.0 (68.4%) and Claude 3.5 (58.7%) are significantly more sensitive when compared to ChatGPT-4o (19.3%). Similar and high performances shown by the LLMs in the non-fracture group indicate that they are competent in detecting normal anatomical structures, but differ in detecting pathological findings. This finding highlights the need for specific training and optimization for the use of LLMs in pediatric radiology. Current performance rates demonstrate that these technologies cannot be used as independent diagnostic tools at this time. However, particularly in urgent circumstances and when access to specialists are limited, LLMs have the potential to be used as assistive tools for assessment and triage. In the future, developing specialized models capable of recognizing anatomical features specific to the pediatric population and evaluating age-specific variations will increase the clinical applicability of these technologies.

## Supporting information

S1 File Supporting information File. (XLSX)

## Abbreviations

DICOM: Digital Imaging and Communications in Medicine
FN: False Negative
FP: False Positive
LLM: Large Language Model
NPV: Negative Predictive Value
PNG: Portable Network Graphic
PPV: Positive Predictive Value
TN: True Negative
TP: True Positive
