# Supplementary material for: Artificial intelligence meets pediatric orthopedics: A comparative analysis of ChatGPT-4o, Gemini 2.0, and Claude 3.5 in detecting supracondylar humeral fractures
Source: PLoS One. 2026 Jul 14;21(7):e0353782. doi: 10.1371/journal.pone.0353782 (PMC13367726; doi:10.1371/journal.pone.0353782)
Supplement: Attachment — Submitted filename: Response to Reviewers.docx [file pone.0353782.s003.docx]

**Response to Reviewers**

**Journal Requirements:**

Reviewer Comment 1:

Please ensure that your manuscript meets PLOS ONE's style requirements, including those for file naming. The PLOS ONE style templates can be found at

https://journals.plos.org/plosone/s/file?id=wjVg/PLOSOne_formatting_sample_main_body.pdf and

<https://journals.plos.org/plosone/s/file?id=ba62/PLOSOne_formatting_sample_title_authors_affiliations.pdf>

Author Response1:

We thank the editorial office for this important note. The manuscript has been thoroughly checked and revised to ensure full compliance with PLOS ONE’s formatting and style requirements, including file naming conventions. We have followed the official templates provided for the main body as well as for the title, authors, and affiliations sections.

Reviewer Comment 2:

Please note that your Data Availability Statement is currently missing the repository name and/or the DOI/accession number of each dataset OR a direct link to access each database. If your manuscript is accepted for publication, you will be asked to provide these details on a very short timeline. We therefore suggest that you provide this information now, though we will not hold up the peer review process if you are unable.

Author Response 2:

We appreciate the reviewer’s comment. The Data Availability Statement has been revised to address the missing details. Relevant information regarding data access has now been provided, and the corresponding data have been uploaded to the system as a supplementary file.

Reviewer Comment 3:

Your ethics statement should only appear in the Methods section of your manuscript. If your ethics statement is written in any section besides the Methods, please delete it from any other section.

Author Response 3:

We thank the editorial office for this clarification. The ethics statement is included only in the Methods section of the manuscript. It does not appear in any other section.

Reviewer Comment 4:

We note that Figure 1 in your submission contain copyrighted images. All PLOS content is published under the Creative Commons Attribution License (CC BY 4.0), which means that the manuscript, images, and Supporting Information files will be freely available online, and any third party is permitted to access, download, copy, distribute, and use these materials in any way, even commercially, with proper attribution. For more information, see our copyright guidelines: http://journals.plos.org/plosone/s/licenses-and-copyright.

We require you to either (1) present written permission from the copyright holder to publish these figures specifically under the CC BY 4.0 license, or (2) remove the figures from your submission:

1. You may seek permission from the original copyright holder of Figure 1 to publish the content specifically under the CC BY 4.0 license.

We recommend that you contact the original copyright holder with the Content Permission Form (http://journals.plos.org/plosone/s/file?id=7c09/content-permission-form.pdf) and the following text:

“I request permission for the open-access journal PLOS ONE to publish XXX under the Creative Commons Attribution License (CCAL) CC BY 4.0 (http://creativecommons.org/licenses/by/4.0/). Please be aware that this license allows unrestricted use and distribution, even commercially, by third parties. Please reply and provide explicit written permission to publish XXX under a CC BY license and complete the attached form.”

Please upload the completed Content Permission Form or other proof of granted permissions as an "Other" file with your submission.

In the figure caption of the copyrighted figure, please include the following text: “Reprinted from [ref] under a CC BY license, with permission from [name of publisher], original copyright [original copyright year].”

2. If you are unable to obtain permission from the original copyright holder to publish these figures under the CC BY 4.0 license or if the copyright holder’s requirements are incompatible with the CC BY 4.0 license, please either i) remove the figure or ii) supply a replacement figure that complies with the CC BY 4.0 license. Please check copyright information on all replacement figures and update the figure caption with source information. If applicable, please specify in the figure caption text when a figure is similar but not identical to the original image and is therefore for illustrative purposes only.

Author Response 4:

We appreciate the reviewer’s careful evaluation and would like to clarify that Figure 1 was created by the authors using artificial intelligence-assisted tools. As the figure is entirely original, no permission is required, and it can be published under the CC BY 4.0 license.

Reviewer Comment5:

We noticed you have some minor occurrence of overlapping text with the following previous publication(s), which needs to be addressed:

http://file.lookus.net/journals/tjtes/bant/v31.i10.pdf

In your revision ensure you cite all your sources (including your own works), and quote or rephrase any duplicated text outside the methods section. Further consideration is dependent on these concerns being addressed.

Author Response5:

We thank the editor for raising this important concern. We carefully reviewed our manuscript for potential text overlap with the referenced publication and other relevant sources. All overlapping text outside the Methods section has been thoroughly revised and rephrased to ensure the originality and clarity of our manuscript.In addition, appropriate citations have been added where necessary, including to our previous related work. We confirm that the current version of the manuscript has been carefully checked to comply with publication ethics standards, and all sources are now properly cited. We believe these revisions have improved the clarity and scientific rigor of the manuscript.

Reviewer Comment 6:

If the reviewer comments include a recommendation to cite specific previously published works, please review and evaluate these publications to determine whether they are relevant and should be cited. There is no requirement to cite these works unless the editor has indicated otherwise.

Author Response 6:

We thank the reviewer for this comment. No specific references were recommended by the reviewers. Nevertheless, we have reviewed the current literature to ensure that relevant and up-to-date studies are appropriately cited in the manuscript. We would be pleased to consider and incorporate any additional relevant references if suggested.

**Response to Reviewers:**

**Reviewer 1:**

AI methods for sc fracture is just tools . This study may add something to literatiure. Au tools are tools for facilitation but in real clinical practice still doubtful and may be not that good to replace the humans . Accuracy with senior surgeon and AI in this respect my be better idea to compare In future

**Response to Reviewers 1:**

We thank the reviewer for this valuable comment and for highlighting the current limitations of artificial intelligence tools in clinical practice. We agree that, at present, AI-based systems should be considered as supportive tools rather than replacements for clinical expertise. In line with this, we have revised the Discussion section to emphasize that large language models are not yet suitable for independent clinical decision-making and should be used as adjunctive tools. We also agree that a direct comparison between AI systems and experienced clinicians would provide important insights. However, such a comparison was beyond the scope of the present study. We have now acknowledged this point as a limitation and suggested it as a direction for future research in the revised manuscript.

**Reviewer 2:**

I appreciate all authors for their nice work in the field of fracture diagnosis with AI tool. I have few concern on confidentiality of data, how they protect data from company using LLMs. Second, hospital having policy to take consent of all patients those are coming to emergency department for data used in research purpose

**Response to Reviewers 2:**

We thank the reviewer for these important comments regarding data confidentiality and patient consent. These aspects were outlined in the Methods section; however, to enhance clarity and transparency, we have further clarified that no personally identifiable or sensitive patient data were entered into the large language models and that all analyses were conducted using fully anonymized data in accordance with data protection principles. We would also like to clarify that ethical approval was obtained and that the requirement for informed consent was waived due to the retrospective design of the study and the use of anonymized data.

**Reviewer 3:**

**Commant 1:**

Methodological Ambiguity regarding "Training" The methods section states: "For more effective interpretation, LLMs were trained using the chapters on supracondylar fractures of humerus in orthopaedic textbooks".
(1)Critique: The term "trained" is technically inaccurate for commercial LLMs accessed via standard interfaces/APIs unless fine-tuning endpoints were used. It appears the authors utilized "In-Context Learning" or "System Prompting."
（2)Requirement: Please replace the term "trained" with "primed" or "provided context." Crucially, the authors must clarify: Was the context window (chat history) cleared/reset for every specific patient? If not, the models likely retained information from previous cases (context leakage), rendering the accuracy data invalid.

**Response** **1:**

We thank the reviewer for this important and insightful comment. We agree that the term “trained” is not technically appropriate in this context. The manuscript has been revised accordingly, and the description has been updated to indicate that the models were provided with contextual information rather than trained.Each case was evaluated in a separate and independent chat session. After each evaluation, the conversation was terminated, and a new session was initiated for the subsequent case. Therefore, no prior interactions were retained between cases, preventing potential context leakage. This has been clarified in the Methods section.

**Commant 2:**

Inadequate Image Resolution The manuscript notes that "Images... were saved in PNG format with a resolution of 512x512 pixels".
（1）Critique: Supracondylar fractures, specifically Gartland Type I, rely on subtle findings such as the posterior fat pad sign or minor cortical disruptions. Downscaling radiographs to 512x512 pixels results in a significant loss of high-frequency detail. Current versions of GPT-4o and Gemini 2.0 support much higher resolution inputs.
（2）Requirement: The authors must justify this preprocessing step. If the study cannot be re-run with high-resolution images, this must be listed as a primary limitation, as it likely handicaps the models' performance on Type I fractures.

**Response** **2:**

We appreciate the reviewer’s valuable suggestion.We acknowledge that the use of 512 × 512 pixel images may limit the detection of subtle radiographic findings, particularly in minimally displaced fractures. This resolution was selected to standardize image inputs and ensure consistent evaluation conditions across all models. However, we agree that this preprocessing step may have affected model performance. Therefore, we have now explicitly acknowledged this as a limitation in the revised manuscript and highlighted the potential benefit of using higher-resolution images in future studies.

**Commant 3.**

Misleading Presentation of "Ideal Accuracy" and Specificity The Abstract states: "All models performed similarly in non-fracture cases with ideal accuracy rates >91%".
（1）Critique: This statement is misleading when contrasted with the results in Table 3, which show that Specificity (TN / TN + FP) is very low, ranging from 33.1% to 36.0%.
（2）Requirement: The "Ideal Accuracy" metric (defined as $\ge 1$ correct response out of 3) obscures the high false-positive rate. In a binary choice, a random guess has a high probability of being correct once in three tries. The Abstract and Discussion must explicitly state that while "Ideal Accuracy" was high, the models exhibited poor Specificity, leading to a high rate of false positives in healthy children.

**Response** 3:

We thank the reviewer for this valuable comment. We agree that the “ideal accuracy” metric may overestimate model performance, particularly in the presence of high false-positive rates. While ideal accuracy rates were high in non-fracture cases, specificity was low across all models, indicating a substantial tendency to incorrectly classify healthy cases as fractures. Accordingly, we have revised the Abstract to explicitly report the low specificity values and have expanded the Discussion to clarify the limitations of this metric and its implications for clinical interpretation.

**Commant 4:**

Lack of Model Specification and Temporal Relevance
（1）Ambiguity of Model Variants: The manuscript generically refers to "Gemini 2.0" and "Claude 3.5". However, these model families typically consist of multiple distinct versions with varying capabilities (e.g., Gemini 2.0 Flash vs. Pro vs. Ultra). Without specifying the exact version, the study is not reproducible.
（2）Rapid Obsolescence: The data collection was performed in May 2025. Given the rapid iteration cycle of LLMs (with newer generations available by early 2026), the findings presented here may already be outdated.
（3）Requirement: The authors must explicitly state the exact model variants used in the Methods section. The Discussion section must acknowledge this time lag as a limitation.

**Response** **4:**

We thank the reviewer for this valuable comment. We agree that precise specification of model variants is essential for reproducibility. Accordingly, we have revised the Methods section to clearly indicate the exact model versions used in this study (ChatGPT-4o Pro, Gemini 2.0 Flash, and Claude 3.5 Pro). We also acknowledge the rapid evolution of large language models. To address this, we have added a statement in the Discussion section noting that the findings reflect model performance at the time of data collection (May 2025) and may not fully represent the capabilities of newer model versions.

**Commant 5:**

Ambiguity in Multi-View Input Strategy The methods mention that "Two-view elbow radiographs were presented", yet the prompt described is singular: "following is the image".
（1）Requirement: Please clarify how two views (AP and Lateral) were input. Were they stitched into a single collage image, or uploaded as two separate files?
Minor Issues
1.Clinical Utility and PPV: Table 3 shows a Positive Predictive Value (PPV) of only 23.2% for ChatGPT-4o. The Discussion should expand on the clinical implications of this: utilizing such a tool would result in a massive number of false positives.
2.Fleiss' Kappa Interpretation: The text reports "good" consistency for ChatGPT-4o in fracture cases ($\kappa=0.69$) but "weak" consistency in non-fracture cases ($\kappa=0.15$). This discrepancy suggests the models are not reliably consistent but are biased towards detecting fractures. Please discuss this distinction.
3.Statistical Reporting: In Table 2, please report exact P-values (e.g., p=0.004) rather than inequalities (p<0.001) where possible.

**Response** **5:**

We thank the reviewer for this helpful comment. We agree that the description of the multi-view image input strategy was not sufficiently clear. In our study, the anteroposterior (AP) and lateral radiographs were provided to the models as separate image files, not as a stitched or combined image. This clarification has now been added to the Methods section. We agree that the low positive predictive value (PPV), particularly for ChatGPT-4o, has important clinical implications. To address this, we have expanded the Discussion section to highlight that the high false-positive rate may lead to unnecessary imaging, overdiagnosis, and increased workload in emergency settings, thereby limiting the clinical utility of these models. We agree that the discrepancy in Fleiss’ kappa values between fracture and non-fracture cases suggests a bias in model behavior. To address this, we have expanded the Discussion section to clarify that the models showed greater consistency in detecting fractures than in identifying normal cases, indicating a tendency to overpredict fractures and contributing to the observed high false-positive rates. Exact P-values have been reported in Table 2 for all values above 0.001. For very small values (p<0.001), inequality notation was retained in accordance with standard statistical reporting practices.

**Reviewer 4:**

**Commants:**

1) Single-center data restricts generalizability—external validation with diverse pediatric populations is essential. 2) No details on LLM fine-tuning protocols (e.g., textbook training scope) limit reproducibility. 3) Lack of head-to-head comparison with radiologists/emergency physicians fails to contextualize LLM performance against clinical gold standards. 4) The 512×512 PNG image resolution may compromise diagnostic detail; DICOM format analysis is recommended. Minor issues include inconsistent Kappa interpretation (κ≥0.61 labeled “very good” but results described as “good”) and a typo (ChatGPT-40 for 4o). Addressing these gaps will strengthen the study’s clinical relevance and methodological rigor, enhancing its value for guiding pediatric AI model development.

**Response to Reviewers 4:** We thank the reviewer for the positive and constructive evaluation of our study. We appreciate the recognition of the novelty and methodological strengths of our work. We have carefully addressed all comments and revised the manuscript accordingly to improve clarity, methodological transparency, and clinical relevance.

1. We thank the reviewer for this important comment. We agree that the single-center design may limit the generalizability of our findings. This has been addressed in the Discussion section. In addition, we have expanded this section to further emphasize that differences in imaging protocols, patient demographics, and disease presentation across institutions may influence model performance. We also highlight the need for multicenter studies to validate the robustness and clinical applicability of these models.
2. We agree that the initial description may have led to ambiguity regarding model training and reproducibility. No fine-tuning or model retraining was performed in this study. Instead, the models were provided with contextual information based on orthopedic textbook content within the input prompts. The Methods section has been revised accordingly to clarify this and to ensure methodological transparency.
3. We thank the reviewer for this important and insightful comment. We agree that the absence of a direct head-to-head comparison with clinicians represents a limitation in contextualizing model performance against clinical gold standards. Although the reference standard in our study was determined by experienced clinicians, we acknowledge that direct performance comparisons would provide more clinically meaningful insights. This point has been addressed in the Discussion section, and we emphasize the need for future studies incorporating such comparisons to better assess clinical applicability.
4. We thank the reviewer for this important and constructive comment. We agree that image resolution is a critical factor in radiographic interpretation. In this study, images were standardized to 512 × 512 pixels to ensure uniform input across models. However, we acknowledge that this preprocessing step may have reduced the visibility of subtle radiographic findings, particularly in minimally displaced (Gartland Type I) fractures. This limitation has been explicitly addressed in the Discussion section. We also highlight that future studies utilizing higher-resolution images or native DICOM formats may improve diagnostic performance and better reflect real-world clinical conditions.

We thank the reviewer for these helpful comments. The interpretation of Fleiss’ Kappa values has been revised to ensure consistency throughout the manuscript, and values ≥0.61 are now described as “very good.” In addition, the typographical error (ChatGPT-40) has been corrected.

**Reviewer 5:**

**Major Comments**

**Commant 1:**

 The manuscript refers to the study as a “systematic review,” which is incorrect. This is a retrospective diagnostic performance study and should be described consistently as such.

**Response** **1:**

We thank the reviewer for this important comment. We agree that the term “systematic review” was inappropriate for this study. The manuscript has been revised accordingly, and the study is now described consistently as a retrospective diagnostic performance study. In particular, the relevant statements in the Introduction have been corrected to reflect the actual study design.

**Commant 2:**

The Methods state that the LLMs were “trained” using textbook chapters. It is unclear whether this refers to fine-tuning or structured prompting. The authors should clearly describe the exact prompts, instructions, and settings used for each model to ensure reproducibility.

**Response** **2:**

We thank the reviewer for this important and constructive comment. We agree that the initial description may have led to ambiguity regarding model training and prompting procedures. No fine-tuning or model retraining was performed in this study. Instead, the models were provided with contextual information through standardized input prompts. We have revised the Methods section to clearly describe the prompt structure, including the clinical scenario and classification instructions, ensuring improved methodological transparency and reproducibility.

**Commant3:**

 Each case was presented three times. The manuscript should clarify:
Whether the unit of analysis is case-level or session-level
Which decision rule (overall/strict/ideal accuracy) was used to derive sensitivity and specificity
There appears to be inconsistency between reported high “ideal accuracy” in non-fracture cases and low specificity values. This should be resolved and clearly explained.

**Response** **3:**

We thank the reviewer for this important and insightful comment. We agree that clarification regarding the unit of analysis and the derivation of diagnostic performance metrics was necessary. In the revised manuscript, we have clarified that the unit of analysis for sensitivity and specificity calculations was defined at the session (response) level rather than the case level. Each case was evaluated three times, and each response was treated as an independent observation for the calculation of TP, FP, TN, and FN values. In contrast, overall accuracy, strict accuracy, and ideal accuracy metrics were calculated at the case level using predefined aggregation rules (all three correct, at least two correct, and at least one correct, respectively). We have also revised the Methods and Discussion sections to explicitly explain this distinction. Regarding the apparent inconsistency between high ideal accuracy in non-fracture cases and low specificity values, this difference arises from the definitions of the metrics. Ideal accuracy is a permissive, case-level metric based on at least one correct response among three attempts, which increases the likelihood of a correct classification. In contrast, specificity was calculated at the response level and reflects the high rate of false-positive responses across repeated sessions. This methodological difference explains the observed discrepancy, and this has now been clearly clarified in the revised manuscript.

**Commant 4:**

The manuscript would benefit from a clearer comparison with previously published non-LLM, image-specific deep learning models (e.g., CNN-based fracture detection systems). Several prior studies using convolutional neural networks or dedicated radiographic AI models have reported substantially higher sensitivity and specificity in musculoskeletal fracture detection tasks. The Discussion should: Compare the reported performance of LLMs (e.g., sensitivity ~68% for Gemini, specificity ~33%) with previously published CNN-based models. Clarify whether LLMs are intended as general-purpose multimodal systems or substitutes for specialized radiology AI.

**Response** **4:**

We thank the reviewer for this important and insightful comment. We agree that a clearer comparison with previously published CNN-based fracture detection models would strengthen the clinical context of our findings. Accordingly, we have expanded the Discussion section to compare the performance of LLMs with that of dedicated radiographic AI models, citing both meta-analytic evidence and clinically validated systems. We also explain that CNN-based models achieve higher diagnostic performance due to their task-specific training on large annotated imaging datasets. Furthermore, we have clarified that LLMs should not be considered as substitutes for specialized radiology AI systems, but rather as general-purpose multimodal tools that may support clinical decision-making.

**Commant 5:**

Discuss why general LLMs may underperform compared to task-specific deep learning models trained on large annotated imaging datasets. This comparison is important to position the clinical relevance of LLM-based image interpretation within the broader AI literature.

**Response** **5:**

We thank the reviewer for this important comment. We agree that further clarification regarding why general-purpose LLMs may underperform compared to task-specific deep learning models was needed. Accordingly, we have expanded the Discussion section to explain that CNN-based models are trained on large, annotated radiographic datasets and optimized for visual feature extraction, whereas LLMs are general-purpose multimodal systems without task-specific optimization for radiographic interpretation. This distinction has been clarified to better position the clinical relevance of LLM-based image interpretation within the broader AI literature.

**Commant 6:**

Classification accuracy is reported only among detected fractures. It would be more clinically meaningful to report overall correct classification (correct detection and correct type combined).

**Response** **6:**

We appreciate the reviewer’s valuable comment. We would like to clarify that our study design inherently incorporates both fracture detection and subsequent classification within a sequential diagnostic workflow. Accordingly, both processes were evaluated together. To enhance clarity, we have revised the manuscript to more explicitly emphasize the relationship between detection and classification performance.

**Commant 7:**

Including representative radiographic images (true positives, false positives, false negatives, and examples of each Gartland type) would improve transparency and allow readers to better understand model errors. All images must be fully anonymized and ethically approved for publication.

**Response** **7:**

We appreciate the reviewer’s valuable suggestion. However, according to national regulations and institutional policies in our country, separate approvals are required for the use and publication of patient imaging data. For this study, ethical approval and data use permission were obtained specifically for the use of anonymized images within the artificial intelligence evaluation process. Therefore, we regret that we are unable to include representative radiographic images for publication. We agree that such additions would improve transparency and encourage future studies designed with appropriate permissions to include representative imaging examples.

**Commant 8:**

The current statement suggests data are available within the hospital system. This may not fully comply with PLOS ONE data policies. The authors should clarify whether anonymized derived data can be shared or provide a formal access process.

**Response** **8:**

We thank the reviewer for this important comment. Due to the use of real patient data, raw imaging data cannot be made publicly available in accordance with institutional and ethical regulations. However, anonymized derived data supporting the findings of this study can be made available from the corresponding author upon reasonable request and with appropriate ethical approval.

**Minor Comments**

**Commant 1:**

Correct typographical errors (e.g., “supracondiler,” “NVP” instead of “NPV”).

**Response** **1:**

The identified typographical errors have been corrected throughout the manuscript, including “supracondiler” revised to “supracondylar” and “NVP” corrected to “NPV”.

**Commant 2:**

Clearly define how ambiguous responses (e.g., “possible fracture”) were categorized.

**Response** 2**:**

We appreciate the reviewer’s comment. In our study, the models were prompted to provide clear binary responses (fracture present or absent), and the outputs did not include ambiguous expressions such as “possible fracture” or “cannot rule out fracture.” Therefore, no additional categorization criteria for ambiguous responses were necessary.

**Commant 3:**

Provide 95% confidence intervals for key diagnostic metrics.

**Response** **3:**

We agree that reporting 95% confidence intervals would improve the robustness of the results. In the revised manuscript, we have added a statement indicating that confidence intervals were considered in the interpretation of diagnostic performance. Future studies including more detailed reporting of confidence intervals are warranted.

**Commant 4:**

Ensure consistent terminology throughout the manuscript.

**Response** **4:**

The manuscript has been carefully reviewed to ensure consistent terminology throughout. Standardized terms have been used for key concepts, including “pediatric,” “Fleiss’ kappa coefficient,” and “supracondylar humeral fracture,” to improve clarity and readability.
