# Supplementary material for: Artificial intelligence meets pediatric orthopedics: A comparative analysis of ChatGPT-4o, Gemini 2.0, and Claude 3.5 in detecting supracondylar humeral fractures
Source: PLoS One. 2026 Jul 14;21(7):e0353782. doi: 10.1371/journal.pone.0353782 (PMC13367726; doi:10.1371/journal.pone.0353782)
Supplement: Peer review history

# Peer review: Decision Letter 0

**DOI:** 10.1371/journal.pone.0353782.r001

**Authors:** Uzair Yaqoob

20 Mar 2026

PONE-D-25-59074

Artificial intelligence meets pediatric orthopedics: a comparative analysis of ChatGPT-4o, Gemini 2.0, and Claude 3.5 in detecting supracondylar humeral fractures

PLOS One

Dear Dr. Kalafat,

Thank you for submitting your manuscript to PLOS ONE. After careful consideration, we feel that it has merit but does not fully meet PLOS ONE’s publication criteria as it currently stands. Therefore, we invite you to submit a revised version of the manuscript that addresses the points raised during the review process.

Please submit your revised manuscript by May 02 2026 11:59PM. If you will need more time than this to complete your revisions, please reply to this message or contact the journal office at plosone@plos.org. When you're ready to submit your revision, log on to https://www.editorialmanager.com/pone/ and select the ’submissions Needing Revision' folder to locate your manuscript file.

Please include the following items when submitting your revised manuscript:

- A letter that responds to each point raised by the academic editor and reviewer(s). You should upload this letter as a separate file labeled 'Response to Reviewers'.
- A marked-up copy of your manuscript that highlights changes made to the original version. You should upload this as a separate file labeled 'Revised Manuscript with Track Changes'.
- An unmarked version of your revised paper without tracked changes. You should upload this as a separate file labeled 'Manuscript'.

If you would like to make changes to your financial disclosure, please include your updated statement in your cover letter. Guidelines for resubmitting your figure files are available below the reviewer comments at the end of this letter.

If applicable, we recommend that you deposit your laboratory protocols in protocols.io to enhance the reproducibility of your results. Protocols.io assigns your protocol its own identifier (DOI) so that it can be cited independently in the future. For instructions see: https://journals.plos.org/plosone/s/submission-guidelines#loc-laboratory-protocols. Additionally, PLOS ONE offers an option for publishing peer-reviewed Lab Protocol articles, which describe protocols hosted on protocols.io. Read more information on sharing protocols at https://plos.org/protocols?utm_medium=editorial-email&utm_source=authorletters&utm_campaign=protocols.

We look forward to receiving your revised manuscript.

Kind regards,

Uzair Yaqoob

Academic Editor

PLOS One

Journal Requirements:

When submitting your revision, we need you to address these additional requirements.

1.Please ensure that your manuscript meets PLOS ONE’s style requirements, including those for file naming. The PLOS ONE style templates can be found at

https://journals.plos.org/plosone/s/file?id=wjVg/PLOSOne_formatting_sample_main_body.pdf and

https://journals.plos.org/plosone/s/file?id=ba62/PLOSOne_formatting_sample_title_authors_affiliations.pdf

2. Please note that your Data Availability Statement is currently missing the repository name and/or the DOI/accession number of each dataset OR a direct link to access each database. If your manuscript is accepted for publication, you will be asked to provide these details on a very short timeline. We therefore suggest that you provide this information now, though we will not hold up the peer review process if you are unable.

3. Your ethics statement should only appear in the Methods section of your manuscript. If your ethics statement is written in any section besides the Methods, please delete it from any other section.

4. We note that Figure 1 in your submission contain copyrighted images. All PLOS content is published under the Creative Commons Attribution License (CC BY 4.0), which means that the manuscript, images, and Supporting Information files will be freely available online, and any third party is permitted to access, download, copy, distribute, and use these materials in any way, even commercially, with proper attribution. For more information, see our copyright guidelines: http://journals.plos.org/plosone/s/licenses-and-copyright.

We require you to either (1) present written permission from the copyright holder to publish these figures specifically under the CC BY 4.0 license, or (2) remove the figures from your submission:

1. You may seek permission from the original copyright holder of Figure 1 to publish the content specifically under the CC BY 4.0 license.

We recommend that you contact the original copyright holder with the Content Permission Form (http://journals.plos.org/plosone/s/file?id=7c09/content-permission-form.pdf) and the following text:

“I request permission for the open-access journal PLOS ONE to publish XXX under the Creative Commons Attribution License (CCAL) CC BY 4.0 (http://creativecommons.org/licenses/by/4.0/). Please be aware that this license allows unrestricted use and distribution, even commercially, by third parties. Please reply and provide explicit written permission to publish XXX under a CC BY license and complete the attached form.”

Please upload the completed Content Permission Form or other proof of granted permissions as an "Other" file with your submission.

In the figure caption of the copyrighted figure, please include the following text: “Reprinted from [ref] under a CC BY license, with permission from [name of publisher], original copyright [original copyright year].”

2. If you are unable to obtain permission from the original copyright holder to publish these figures under the CC BY 4.0 license or if the copyright holder’s requirements are incompatible with the CC BY 4.0 license, please either i) remove the figure or ii) supply a replacement figure that complies with the CC BY 4.0 license. Please check copyright information on all replacement figures and update the figure caption with source information. If applicable, please specify in the figure caption text when a figure is similar but not identical to the original image and is therefore for illustrative purposes only.

5. We noticed you have some minor occurrence of overlapping text with the following previous publication(s), which needs to be addressed:

http://file.lookus.net/journals/tjtes/bant/v31.i10.pdf

In your revision ensure you cite all your sources (including your own works), and quote or rephrase any duplicated text outside the methods section. Further consideration is dependent on these concerns being addressed.

6. If the reviewer comments include a recommendation to cite specific previously published works, please review and evaluate these publications to determine whether they are relevant and should be cited. There is no requirement to cite these works unless the editor has indicated otherwise.

[Note: HTML markup is below. Please do not edit.]

Reviewers' comments:

Reviewer’s Responses to Questions

**Comments to the Author**

1. Is the manuscript technically sound, and do the data support the conclusions?

The manuscript must describe a technically sound piece of scientific research with data that supports the conclusions. Experiments must have been conducted rigorously, with appropriate controls, replication, and sample sizes. The conclusions must be drawn appropriately based on the data presented.

Reviewer #1: Yes

Reviewer #2: Yes

Reviewer #3: Partly

Reviewer #4: Yes

Reviewer #5: Yes

**********

2. Has the statistical analysis been performed appropriately and rigorously?

Reviewer #1: Yes

Reviewer #2: Yes

Reviewer #3: No

Reviewer #4: Yes

Reviewer #5: No

**********

3. Have the authors made all data underlying the findings in their manuscript fully available?

The PLOS Data policy requires authors to make all data underlying the findings described in their manuscript fully available without restriction, with rare exception (please refer to the Data Availability Statement in the manuscript PDF file). The data should be provided as part of the manuscript or its supporting information, or deposited to a public repository. For example, in addition to summary statistics, the data points behind means, medians and variance measures should be available. If there are restrictions on publicly sharing data—e.g. participant privacy or use of data from a third party—those must be specified.

Reviewer #1: Yes

Reviewer #2: Yes

Reviewer #3: No

Reviewer #4: Yes

Reviewer #5: Yes

**********

4. Is the manuscript presented in an intelligible fashion and written in standard English?

PLOS ONE does not copyedit accepted manuscripts, so the language in submitted articles must be clear, correct, and unambiguous. Any typographical or grammatical errors should be corrected at revision, so please note any specific errors here.

Reviewer #1: Yes

Reviewer #2: Yes

Reviewer #3: Yes

Reviewer #4: Yes

Reviewer #5: No

**********

5. Review Comments to the Author

Please use the space provided to explain your answers to the questions above. You may also include additional comments for the author, including concerns about dual publication, research ethics, or publication ethics. (Please upload your review as an attachment if it exceeds 20,000 characters)

Reviewer #1: AI methods for sc fracture is just tools . This study may add something to literatiure. Au tools are tools for facilitation but in real clinical practice still doubtful and may be not that good to replace the humans . Accuracy with senior surgeon and AI in this respect my be better idea to compare

In future

Reviewer #2: I appreciate all authors for their nice work in the field of fracture diagnosis with AI tool.

I have few concern on confidentiality of data, how they protect data from company using LLMs.

second, hospital having policy to take consent of all patients those are coming to emergency department for data used in research purpose

Reviewer #3: Manuscript Number: PONE-D-25-59074 Title: Artificial intelligence meets pediatric orthopedics: a comparative analysis of ChatGPT-4o, Gemini 2.0, and Claude 3.5 in detecting supracondylar humeral fractures

General Assessment This study evaluates the performance of three large multimodal models (ChatGPT-4o, Gemini 2.0, and Claude 3.5) in detecting and classifying pediatric supracondylar humeral fractures. The inclusion of the latest models, particularly Gemini 2.0, is a strength and adds value to the current literature. However, there are significant concerns regarding the methodology description, image preprocessing, and the interpretation of statistical results that must be addressed.

Major Issues

1. Methodological Ambiguity regarding "Training" The methods section states: "For more effective interpretation, LLMs were trained using the chapters on supracondylar fractures of humerus in orthopaedic textbooks".

(1)Critique: The term "trained" is technically inaccurate for commercial LLMs accessed via standard interfaces/APIs unless fine-tuning endpoints were used. It appears the authors utilized "In-Context Learning" or "System Prompting."

�2)Requirement: Please replace the term "trained" with "primed" or "provided context." Crucially, the authors must clarify: Was the context window (chat history) cleared/reset for every specific patient? If not, the models likely retained information from previous cases (context leakage), rendering the accuracy data invalid.

2. Inadequate Image Resolution The manuscript notes that "Images... were saved in PNG format with a resolution of 512x512 pixels".

�1�Critique: Supracondylar fractures, specifically Gartland Type I, rely on subtle findings such as the posterior fat pad sign or minor cortical disruptions. Downscaling radiographs to 512x512 pixels results in a significant loss of high-frequency detail. Current versions of GPT-4o and Gemini 2.0 support much higher resolution inputs.

�2�Requirement: The authors must justify this preprocessing step. If the study cannot be re-run with high-resolution images, this must be listed as a primary limitation, as it likely handicaps the models' performance on Type I fractures.

3. Misleading Presentation of "Ideal Accuracy" and Specificity The Abstract states: "All models performed similarly in non-fracture cases with ideal accuracy rates >91%".

�1�Critique: This statement is misleading when contrasted with the results in Table 3, which show that Specificity (TN / TN + FP) is very low, ranging from 33.1% to 36.0%.

�2�Requirement: The "Ideal Accuracy" metric (defined as $\ge 1$ correct response out of 3) obscures the high false-positive rate. In a binary choice, a random guess has a high probability of being correct once in three tries. The Abstract and Discussion must explicitly state that while "Ideal Accuracy" was high, the models exhibited poor Specificity, leading to a high rate of false positives in healthy children.

4. Lack of Model Specification and Temporal Relevance

�1�Ambiguity of Model Variants: The manuscript generically refers to "Gemini 2.0" and "Claude 3.5". However, these model families typically consist of multiple distinct versions with varying capabilities (e.g., Gemini 2.0 Flash vs. Pro vs. Ultra). Without specifying the exact version, the study is not reproducible.

�2�Rapid Obsolescence: The data collection was performed in May 2025. Given the rapid iteration cycle of LLMs (with newer generations available by early 2026), the findings presented here may already be outdated.

�3�Requirement: The authors must explicitly state the exact model variants used in the Methods section. The Discussion section must acknowledge this time lag as a limitation.

5. Ambiguity in Multi-View Input Strategy The methods mention that "Two-view elbow radiographs were presented", yet the prompt described is singular: "following is the image".

�1�Requirement: Please clarify how two views (AP and Lateral) were input. Were they stitched into a single collage image, or uploaded as two separate files?

Minor Issues

1.Clinical Utility and PPV: Table 3 shows a Positive Predictive Value (PPV) of only 23.2% for ChatGPT-4o. The Discussion should expand on the clinical implications of this: utilizing such a tool would result in a massive number of false positives.

2.Fleiss' Kappa Interpretation: The text reports "good" consistency for ChatGPT-4o in fracture cases ($\kappa=0.69$) but "weak" consistency in non-fracture cases ($\kappa=0.15$). This discrepancy suggests the models are not reliably consistent but are biased towards detecting fractures. Please discuss this distinction.

3.Statistical Reporting: In Table 2, please report exact P-values (e.g., p=0.004) rather than inequalities (p<0.001) where possible.

Reviewer #4: This study is the first to compare ChatGPT-4o, Gemini 2.0, and Claude 3.5 for pediatric supracondylar humeral fracture detection and Gartland classification, addressing a critical gap in pediatric radiology AI research. The retrospective design with 300 well-characterized patients, rigorous accuracy metrics (overall/strict/ideal), and consistency analysis via Fleiss’ Kappa are major strengths, and the conclusion linking model performance to pediatric-specific training needs is well-supported by results.

However, several limitations warrant revision: 1) Single-center data restricts generalizability—external validation with diverse pediatric populations is essential. 2) No details on LLM fine-tuning protocols (e.g., textbook training scope) limit reproducibility. 3) Lack of head-to-head comparison with radiologists/emergency physicians fails to contextualize LLM performance against clinical gold standards. 4) The 512×512 PNG image resolution may compromise diagnostic detail; DICOM format analysis is recommended.

Minor issues include inconsistent Kappa interpretation (κ≥0.61 labeled “very good” but results described as “good”) and a typo (ChatGPT-40 for 4o). Addressing these gaps will strengthen the study’s clinical relevance and methodological rigor, enhancing its value for guiding pediatric AI model development.

Reviewer #5: Summary

This study evaluates the performance of three large language models (ChatGPT-4o, Gemini 2.0, and Claude 3.5) in detecting pediatric supracondylar humeral fractures and classifying them using the modified Gartland system. The topic is timely and clinically relevant. The sample size is adequate, and the assessment of response consistency across repeated sessions is a strength. The comparative evaluation of multiple multimodal LLMs adds value to the current literature.

However, several methodological clarifications and contextual comparisons are necessary before publication.

Major Comments

1.The manuscript refers to the study as a “systematic review,” which is incorrect. This is a retrospective diagnostic performance study and should be described consistently as such.

2. The Methods state that the LLMs were “trained” using textbook chapters. It is unclear whether this refers to fine-tuning or structured prompting. The authors should clearly describe the exact prompts, instructions, and settings used for each model to ensure reproducibility.

3. Each case was presented three times. The manuscript should clarify:

Whether the unit of analysis is case-level or session-level

Which decision rule (overall/strict/ideal accuracy) was used to derive sensitivity and specificity

There appears to be inconsistency between reported high “ideal accuracy” in non-fracture cases and low specificity values. This should be resolved and clearly explained.

4. The manuscript would benefit from a clearer comparison with previously published non-LLM, image-specific deep learning models (e.g., CNN-based fracture detection systems). Several prior studies using convolutional neural networks or dedicated radiographic AI models have reported substantially higher sensitivity and specificity in musculoskeletal fracture detection tasks. The Discussion should: Compare the reported performance of LLMs (e.g., sensitivity ~68% for Gemini, specificity ~33%) with previously published CNN-based models. Clarify whether LLMs are intended as general-purpose multimodal systems or substitutes for specialized radiology AI.

5. Discuss why general LLMs may underperform compared to task-specific deep learning models trained on large annotated imaging datasets. This comparison is important to position the clinical relevance of LLM-based image interpretation within the broader AI literature.

6. Classification accuracy is reported only among detected fractures. It would be more clinically meaningful to report overall correct classification (correct detection and correct type combined).

7. Including representative radiographic images (true positives, false positives, false negatives, and examples of each Gartland type) would improve transparency and allow readers to better understand model errors. All images must be fully anonymized and ethically approved for publication.

8. The current statement suggests data are available within the hospital system. This may not fully comply with PLOS ONE data policies. The authors should clarify whether anonymized derived data can be shared or provide a formal access process.

Minor Comments

1. Correct typographical errors (e.g., “supracondiler,” “NVP” instead of “NPV”).

2. Clearly define how ambiguous responses (e.g., “possible fracture”) were categorized.

3. Provide 95% confidence intervals for key diagnostic metrics.

4. Ensure consistent terminology throughout the manuscript.

**********

6. PLOS authors have the option to publish the peer review history of their article (what does this mean?). If published, this will include your full peer review and any attached files.

If you choose “no”, your identity will remain anonymous but your review may still be made public.

**Do you want your identity to be public for this peer review?**  For information about this choice, including consent withdrawal, please see our Privacy Policy.

Reviewer #1: **Yes:** Nio

Reviewer #2: **Yes:** Irfan M Rajput, Consultant Orthopedic and Sports Surgeon at Dow university Health Sciences Karachi

Reviewer #3: No

Reviewer #4: **Yes:** Tianlun Zhang

Reviewer #5: **Yes:** Chihiro Tanikawa

**********

[NOTE: If reviewer comments were submitted as an attachment file, they will be attached to this email and accessible via the submission site. Please log into your account, locate the manuscript record, and check for the action link "View Attachments". If this link does not appear, there are no attachment files.]

To ensure your figures meet our technical requirements, please review our figure guidelines: https://journals.plos.org/plosone/s/figures

You may also use PLOS’s free figure tool, NAAS, to help you prepare publication quality figures: https://journals.plos.org/plosone/s/figures#loc-tools-for-figure-preparation.

NAAS will assess whether your figures meet our technical requirements by comparing each figure against our figure specifications.

# Author comment: Author response to Decision Letter 1

**DOI:** 10.1371/journal.pone.0353782.r002

14 Apr 2026

Response to Reviewers

Journal Requirements:

Reviewer Comment 1:

Please ensure that your manuscript meets PLOS ONE’s style requirements, including those for file naming. The PLOS ONE style templates can be found at

https://journals.plos.org/plosone/s/file?id=wjVg/PLOSOne_formatting_sample_main_body.pdf and

https://journals.plos.org/plosone/s/file?id=ba62/PLOSOne_formatting_sample_title_authors_affiliations.pdf

Author Response1:

We thank the editorial office for this important note. The manuscript has been thoroughly checked and revised to ensure full compliance with PLOS ONE’s formatting and style requirements, including file naming conventions. We have followed the official templates provided for the main body as well as for the title, authors, and affiliations sections.

Reviewer Comment 2:

Please note that your Data Availability Statement is currently missing the repository name and/or the DOI/accession number of each dataset OR a direct link to access each database. If your manuscript is accepted for publication, you will be asked to provide these details on a very short timeline. We therefore suggest that you provide this information now, though we will not hold up the peer review process if you are unable.

Author Response 2:

We appreciate the reviewer’s comment. The Data Availability Statement has been revised to address the missing details. Relevant information regarding data access has now been provided, and the corresponding data have been uploaded to the system as a supplementary file.

Reviewer Comment 3:

Your ethics statement should only appear in the Methods section of your manuscript. If your ethics statement is written in any section besides the Methods, please delete it from any other section.

Author Response 3:

We thank the editorial office for this clarification. The ethics statement is included only in the Methods section of the manuscript. It does not appear in any other section.

Reviewer Comment 4:

We note that Figure 1 in your submission contain copyrighted images. All PLOS content is published under the Creative Commons Attribution License (CC BY 4.0), which means that the manuscript, images, and Supporting Information files will be freely available online, and any third party is permitted to access, download, copy, distribute, and use these materials in any way, even commercially, with proper attribution. For more information, see our copyright guidelines: http://journals.plos.org/plosone/s/licenses-and-copyright.

We require you to either (1) present written permission from the copyright holder to publish these figures specifically under the CC BY 4.0 license, or (2) remove the figures from your submission:

1. You may seek permission from the original copyright holder of Figure 1 to publish the content specifically under the CC BY 4.0 license.

We recommend that you contact the original copyright holder with the Content Permission Form (http://journals.plos.org/plosone/s/file?id=7c09/content-permission-form.pdf) and the following text:

“I request permission for the open-access journal PLOS ONE to publish XXX under the Creative Commons Attribution License (CCAL) CC BY 4.0 (http://creativecommons.org/licenses/by/4.0/). Please be aware that this license allows unrestricted use and distribution, even commercially, by third parties. Please reply and provide explicit written permission to publish XXX under a CC BY license and complete the attached form.”

Please upload the completed Content Permission Form or other proof of granted permissions as an "Other" file with your submission.

In the figure caption of the copyrighted figure, please include the following text: “Reprinted from [ref] under a CC BY license, with permission from [name of publisher], original copyright [original copyright year].”

2. If you are unable to obtain permission from the original copyright holder to publish these figures under the CC BY 4.0 license or if the copyright holder’s requirements are incompatible with the CC BY 4.0 license, please either i) remove the figure or ii) supply a replacement figure that complies with the CC BY 4.0 license. Please check copyright information on all replacement figures and update the figure caption with source information. If applicable, please specify in the figure caption text when a figure is similar but not identical to the original image and is therefore for illustrative purposes only.

Author Response 4:

We appreciate the reviewer’s careful evaluation and would like to clarify that Figure 1 was created by the authors using artificial intelligence-assisted tools. As the figure is entirely original, no permission is required, and it can be published under the CC BY 4.0 license.

Reviewer Comment5:

We noticed you have some minor occurrence of overlapping text with the following previous publication(s), which needs to be addressed:

http://file.lookus.net/journals/tjtes/bant/v31.i10.pdf

In your revision ensure you cite all your sources (including your own works), and quote or rephrase any duplicated text outside the methods section. Further consideration is dependent on these concerns being addressed.

Author Response5:

We thank the editor for raising this important concern. We carefully reviewed our manuscript for potential text overlap with the referenced publication and other relevant sources. All overlapping text outside the Methods section has been thoroughly revised and rephrased to ensure the originality and clarity of our manuscript.In addition, appropriate citations have been added where necessary, including to our previous related work. We confirm that the current version of the manuscript has been carefully checked to comply with publication ethics standards, and all sources are now properly cited. We believe these revisions have improved the clarity and scientific rigor of the manuscript.

Reviewer Comment 6:

If the reviewer comments include a recommendation to cite specific previously published works, please review and evaluate these publications to determine whether they are relevant and should be cited. There is no requirement to cite these works unless the editor has indicated otherwise.

Author Response 6:

We thank the reviewer for this comment. No specific references were recommended by the reviewers. Nevertheless, we have reviewed the current literature to ensure that relevant and up-to-date studies are appropriately cited in the manuscript. We would be pleased to consider and incorporate any additional relevant references if suggested.

Response to Reviewers:

Reviewer 1:

AI methods for sc fracture is just tools . This study may add something to literatiure. Au tools are tools for facilitation but in real clinical practice still doubtful and may be not that good to replace the humans . Accuracy with senior surgeon and AI in this respect my be better idea to compare In future

Response to Reviewers 1:

We thank the reviewer for this valuable comment and for highlighting the current limitations of artificial intelligence tools in clinical practice. We agree that, at present, AI-based systems should be considered as supportive tools rather than replacements for clinical expertise. In line with this, we have revised the Discussion section to emphasize that large language models are not yet suitable for independent clinical decision-making and should be used as adjunctive tools. We also agree that a direct comparison between AI systems and experienced clinicians would provide important insights. However, such a comparison was beyond the scope of the present study. We have now acknowledged this point as a limitation and suggested it as a direction for future research in the revised manuscript.

Reviewer 2:

I appreciate all authors for their nice work in the field of fracture diagnosis with AI tool. I have few concern on confidentiality of data, how they protect data from company using LLMs. Second, hospital having policy to take consent of all patients those are coming to emergency department for data used in research purpose

Response to Reviewers 2:

We thank the reviewer for these important comments regarding data confidentiality and patient consent. These aspects were outlined in the Methods section; however, to enhance clarity and transparency, we have further clarified that no personally identifiable or sensitive patient data were entered into the large language models and that all analyses were conducted using fully anonymized data in accordance with data protection principles. We would also like to clarify that ethical approval was obtained and that the requirement for informed consent was waived due to the retrospective design of the study and the use of anonymized data.

Reviewer 3:

Commant 1:

Methodological Ambiguity regarding "Training" The methods section states: "For more effective interpretation, LLMs were trained using the chapters on supracondylar fractures of humerus in orthopaedic textbooks".

(1)Critique: The term "trained" is technically inaccurate for commercial LLMs accessed via standard interfaces/APIs unless fine-tuning endpoints were used. It appears the authors utilized "In-Context Learning" or "System Prompting."

�2)Requirement: Please replace the term "trained" with "primed" or "provided context." Crucially, the authors must clarify: Was the context window (chat history) cleared/reset for every specific patient? If not, the models likely retained information from previous cases (context leakage), rendering the accuracy data invalid.

Response 1:

We thank the reviewer for this important and insightful comment. We agree that the term “trained” is not technically appropriate in this context. The manuscript has been revised accordingly, and the description has been updated to indicate that the models were provided with contextual information rather than trained.Each case was evaluated in a separate and independent chat session. After each evaluation, the conversation was terminated, and a new session was initiated for the subsequent case. Therefore, no prior interactions were retained between cases, preventing potential context leakage. This has been clarified in the Methods section.

Commant 2:

Inadequate Image Resolution The manuscript notes that "Images... were saved in PNG format with a resolution of 512x512 pixels".

�1�Critique: Supracondylar fractures, specifically Gartland Type I, rely on subtle findings such as the posterior fat pad sign or minor cortical disruptions. Downscaling radiographs to 512x512 pixels results in a significant loss of high-frequency detail. Current versions of GPT-4o and Gemini 2.0 support much higher resolution inputs.

�2�Requirement: The authors must justify this preprocessing step. If the study cannot be re-run with high-resolution images, this must be listed as a primary limitation, as it likely handicaps the models' performance on Type I fractures.

Response 2:

We appreciate the reviewer’s valuable suggestion.We acknowledge that the use of 512 × 512 pixel images may limit the detection of subtle radiographic findings, particularly in minimally displaced fractures. This resolution was selected to standardize image inputs and ensure consistent evaluation conditions across all models. However, we agree that this preprocessing step may have affected model performance. Therefore, we have now explicitly acknowledged this as a limitation in the revised manuscript and highlighted the potential benefit of using higher-resolution images in future studies.

Commant 3.

Misleading Presentation of "Ideal Accuracy" and Specificity The Abstract states: "All models performed similarly in non-fracture cases with ideal accuracy rates >91%".

�1�Critique: This statement is misleading when contrasted with the results in Table 3, which show that Specificity (TN / TN + FP) is very low, ranging from 33.1% to 36.0%.

�2�Requirement: The "Ideal Accuracy" metric (defined as $\ge 1$ correct response out of 3) obscures the high false-positive rate. In a binary choice, a random guess has a high probability of being correct once in three tries. The Abstract and Discussion must explicitly state that while "Ideal Accuracy" was high, the models exhibited poor Specificity, leading to a high rate of false positives in healthy children.

Response 3:

We thank the reviewer for this valuable comment. We agree that the “ideal accuracy” metric may overestimate model performance, particularly in the presence of high false-positive rates. While ideal accuracy rates were high in non-fracture cases, specificity was low across all models, indicating a substantial tendency to incorrectly classify healthy cases as fractures. Accordingly, we have revised the Abstract to explicitly report the low specificity values and have expanded the Discussion to clarify the limitations of this metric and its implications for clinical interpretation.

Commant 4:

Lack of Model Specification and Temporal Relevance

�1�Ambiguity of Model Variants: The manuscript generically refers to "Gemini 2.0" and "Claude 3.5". However, these model families typically consist of multiple distinct versions with varying capabilities (e.g., Gemini 2.0 Flash vs. Pro vs. Ultra). Without specifying the exact version, the study is not reproducible.

�2�Rapid Obsolescence: The data collection was performed in May 2025. Given the rapid iteration cycle of LLMs (with newer generations available by early 2026), the findings presented here may already be outdated.

�3�Requirement: The authors must explicitly state the exact model variants used in the Methods section. The Discussion section must acknowledge this time lag as a limitation.

Response 4:

We thank the reviewer for this valuable comment. We agree that precise specification of model variants is essential for reproducibility. Accordingly, we have revised the Methods section to clearly indicate the exact model versions used in this study (ChatGPT-4o Pro, Gemini 2.0 Flash, and Claude 3.5 Pro). We also acknowledge the rapid evolution of large language models. To address this, we have added a statement in the Discussion section noting that the findings reflect model performance at the time of data collection (May 2025) and may not fully represent the capabilities of newer model versions.

Commant 5:

Ambiguity in Multi-View Input Strategy The methods mention that "Two-view elbow radiographs were presented", yet the prompt described is singular: "following is the image".

�1�Requirement: Please clarify how two views (AP and Lateral) were input. Were they stitched into a single collage image, or uploaded as two separate files?

Minor Issues

1.Clinical Utility and PPV: Table 3 shows a Positive Predictive Value (PPV) of only 23.2% for ChatGPT-4o. The Discussion should expand on the clinical implications of this: utilizing such a tool would result in a massive number of false positives.

2.Fleiss' Kappa Interpretation: The text reports "good" consistency for ChatGPT-4o in fracture cases ($\kappa=0.69$) but "weak" consistency in non-fracture cases ($\kappa=0.15$). This discrepancy suggests the models are not reliably consistent but are biased towards detecting fractures. Please discuss this distinction.

3.Statistical Reporting: In Table 2, please report exact P-values (e.g., p=0.004) rather than inequalities (p<0.001) where possible.

Response 5:

We thank the reviewer for this helpful comment. We agree that the description of the multi-view image input strategy was not sufficiently clear. In our study, the anteroposterior (AP) and lateral radiographs were provided to the models as separate image files, not as a stitched or combined image. This clarification has now been added to the Methods section. We agree that the low positive predictive value (PPV), particularly for ChatGPT-4o, has important clinical implications. To address this, we have expanded the Discussion section to highlight that the high false-positive rate may lead to unnecessary imaging, overdiagnosis, and increased workload in emergency settings, thereby limiting the clinical utility of these models. We agree that the discrepancy in Fleiss’ kappa values between fracture and non-fracture cases suggests a bias in model behavior. To address this, we have expanded the Discussion section to clarify that the models sho

Attachment Submitted filename: Response to Reviewers.docx

# Peer review: Decision Letter 1

**DOI:** 10.1371/journal.pone.0353782.r003

**Authors:** Lisong Zhang

29 Jun 2026

Artificial intelligence meets pediatric orthopedics: a comparative analysis of ChatGPT-4o, Gemini 2.0, and Claude 3.5 in detecting supracondylar humeral fractures

PONE-D-25-59074R1

Dear Dr. Kalafat,

We’re pleased to inform you that your manuscript has been judged scientifically suitable for publication and will be formally accepted for publication once it meets all outstanding technical requirements.

Within one week, you’ll receive an e-mail detailing the required amendments. When these have been addressed, you’ll receive a formal acceptance letter and your manuscript will be scheduled for publication.

An invoice will be generated when your article is formally accepted. Please note, if your institution has a publishing partnership with PLOS and your article meets the relevant criteria, all or part of your publication costs will be covered. Please make sure your user information is up-to-date by logging into Editorial Manager at Editorial Manager® and clicking the ‘Update My Information' link at the top of the page. For questions related to billing, please contact billing support.

If your institution or institutions have a press office, please notify them about your upcoming paper to help maximize its impact. If they’ll be preparing press materials, please inform our press team as soon as possible -- no later than 48 hours after receiving the formal acceptance. Your manuscript will remain under strict press embargo until 2 pm Eastern Time on the date of publication. For more information, please contact onepress@plos.org.

Kind regards,

Lisong Zhang

Academic Editor

PLOS One

Additional Editor Comments (optional):

Reviewer 3

AI methods for sc fracture is just tools . This study may add something to literatiure. Au tools are tools for facilitation but in real clinical practice still doubtful and may be not that good to replace the humans . Accuracy with senior surgeon and AI in this respect my be better idea to compare

In future

Reviewers' comments:

Reviewer’s Responses to Questions

**Comments to the Author**

1. If the authors have adequately addressed your comments raised in a previous round of review and you feel that this manuscript is now acceptable for publication, you may indicate that here to bypass the “Comments to the Author” section, enter your conflict of interest statement in the “Confidential to Editor” section, and submit your "Accept" recommendation.

Reviewer #3: All comments have been addressed

**********

2. Is the manuscript technically sound, and do the data support the conclusions?

The manuscript must describe a technically sound piece of scientific research with data that supports the conclusions. Experiments must have been conducted rigorously, with appropriate controls, replication, and sample sizes. The conclusions must be drawn appropriately based on the data presented.

Reviewer #3: Yes

**********

3. Has the statistical analysis been performed appropriately and rigorously?

Reviewer #3: Yes

**********

4. Have the authors made all data underlying the findings in their manuscript fully available?

The PLOS Data policy requires authors to make all data underlying the findings described in their manuscript fully available without restriction, with rare exception (please refer to the Data Availability Statement in the manuscript PDF file). The data should be provided as part of the manuscript or its supporting information, or deposited to a public repository. For example, in addition to summary statistics, the data points behind means, medians and variance measures should be available. If there are restrictions on publicly sharing data—e.g. participant privacy or use of data from a third party—those must be specified.

Reviewer #3: Yes

**********

5. Is the manuscript presented in an intelligible fashion and written in standard English?

PLOS ONE does not copyedit accepted manuscripts, so the language in submitted articles must be clear, correct, and unambiguous. Any typographical or grammatical errors should be corrected at revision, so please note any specific errors here.

Reviewer #3: Yes

**********

6. Review Comments to the Author

Please use the space provided to explain your answers to the questions above. You may also include additional comments for the author, including concerns about dual publication, research ethics, or publication ethics. (Please upload your review as an attachment if it exceeds 20,000 characters)

Reviewer #3: Manuscript Number: PONE-D-25-59074R1

Title: Artificial intelligence meets pediatric orthopedics: a comparative analysis of ChatGPT-4o, Gemini 2.0, and Claude 3.5 in detecting supracondylar humeral fractures

The revised manuscript and the accompanying response letter have been thoroughly reviewed. The authors have provided satisfactory and scientifically rigorous responses to all major critiques.

Specifically, the methodological transparency achieved by clarifying the independent chat session protocol effectively eliminates prior concerns regarding context leakage. Furthermore, the objective reporting of specificity in the revised Abstract, alongside the explicit acknowledgment of image resolution constraints (512x512 pixels) and precise model versioning in the Discussion, significantly enhances the academic rigor and reproducibility of the paper.

The manuscript now meets the necessary methodological and reporting standards. No further revisions are required.

**********

7. PLOS authors have the option to publish the peer review history of their article (what does this mean?). If published, this will include your full peer review and any attached files.

If you choose “no”, your identity will remain anonymous but your review may still be made public.

**Do you want your identity to be public for this peer review?**  For information about this choice, including consent withdrawal, please see our Privacy Policy.

Reviewer #3: No

**********

# Editor report: Acceptance letter

**DOI:** 10.1371/journal.pone.0353782.r004

**Authors:** Lisong Zhang

PONE-D-25-59074R1

PLOS One

Dear Dr. Kalafat,

I'm pleased to inform you that your manuscript has been deemed suitable for publication in PLOS One. Congratulations! Your manuscript is now being handed over to our production team.

At this stage, our production department will prepare your paper for publication. This includes ensuring the following:

* All references, tables, and figures are properly cited

* All relevant supporting information is included in the manuscript submission,

* There are no issues that prevent the paper from being properly typeset

You will receive further instructions from the production team, including instructions on how to review your proof when it is ready. Please keep in mind that we are working through a large volume of accepted articles, so please give us a few days to review your paper and let you know the next and final steps.

Lastly, if your institution or institutions have a press office, please let them know about your upcoming paper now to help maximize its impact. If they'll be preparing press materials, please inform our press team within the next 48 hours. Your manuscript will remain under strict press embargo until 2 pm Eastern Time on the date of publication. For more information, please contact onepress@plos.org.

You will receive an invoice from PLOS for your publication fee after your manuscript has reached the completed accept phase. If you receive an email requesting payment before acceptance or for any other service, this may be a phishing scheme. Learn how to identify phishing emails and protect your accounts at https://explore.plos.org/phishing.

If we can help with anything else, please email us at customercare@plos.org.

Thank you for submitting your work to PLOS ONE and supporting open access.

Kind regards,

PLOS ONE Editorial Office Staff

on behalf of

Associate Professor Lisong Zhang

Academic Editor

PLOS One
